# Prognostic significance of high NPC2 expression in gastric cancer

**DOI:** 10.1038/s41598-023-47882-3

**Published:** 2023-11-24

**Authors:** Yunzhuang Yao, Jinnan Ren, Junhui Lu, Yue Sui, Jingwen Gong, Xing Chen

**Affiliations:** 1 Department of Gastroenterology, First Hospital of Shanxi Medical University, Taiyuan, 030000 China; 2 Faculty of Graduate Studies, Shanxi Medical University, Taiyuan, 030000 China; 3 Heping Hospital Affiliated to Changzhi Medical College, Changzhi, China

**Keywords:** Gastrointestinal cancer, Prognostic markers

## Abstract

Gastric cancer is one of the most common malignancies worldwide, and the third leading cause of cancer-related death. The identification of novel biomarkers and therapeutic targets is critical to improve the prognosis. A total of 380 patients with primary gastric cancer from the TCGA database were analyzed. The receiver operating characteristic curves were plotted. We further evaluated the independent prognostic ability of NPC2 expression for overall survival (OS) and relapse-free survival (RFS) through the Kaplan–Meier curve and Cox analysis. The NPC2 expression was significantly higher (*P* < 0.001) in gastric cancer. High NPC2 expression was significantly (*P* < 0.0001) associated with poor OS and poor RFS. The age, stage, radiation therapy, residual tumor, and NPC2 expression showed independent prognostic value for OS. The gender and NPC2 expression showed independent prognostic value for RFS. The higher NPC2 expression was observed in gastric cancer, compared with adjacent normal tissue (*P* < 0.001), confirmed by the IHC staining. The CCK-8 assay showed that NPC2 knockdown inhibits cell proliferation while NPC2 overexpression promotes cell proliferation (*P* < 0.05). NPC2 expression may serve as a promising prognostic biomarker for patients with gastric cancer.

## Introduction

Gastric cancer, originated from gastric mucosal epithelium, is one of the most common malignancies worldwide threatening human health^1^. Gastric cancer is the third leading cause of cancer-related death with more than 1 million new cases each year^2^. Due to the limited understanding of molecular mechanisms and poor prognosis, the mortality rate of gastric cancer is still high^3^. Gastric cancer has an insidious onset and atypical early symptoms are often not appreciated by patients^4^. The majority of patients are found to have advanced gastric cancer, and their 5-year survival rates tend to be less than 10%^5^. Therefore, identification of novel biomarkers and therapeutic targets is critical to improve the diagnosis, treatment and prognosis of gastric cancer^6, 7^.

NPC2 (NPC intracellular cholesterol transporter 2) is an intracellular cholesterol transporting protein that affects the cholesterol homeostasis regulatory system^8, 9^. In recent years, increasing evidence has revealed the important role of NPC2 in the development and progression of various human cancers, including gastric cancer^10^. There is a very strong association between NPC2 expression and tumors, with a signature of overexpression in breast, colon, as well as lung cancers^11^. However, the clinical significance and prognostic value of NPC2 expression in gastric cancer remain largely unknown.

The Cancer Genome Atlas (TCGA) is a comprehensive and integrated database that provides a wealth of genomic, transcriptomic, and clinical information on multiple types of cancer, including gastric cancer^12, 13^. In this study, we aimed to assess the correlation between NPC2 expression in gastric cancer and clinicopathologic characteristics through analyzing data from the TCGA database. The receiver operating characteristic (ROC) curves were plotted to analyze the diagnostic value of NPC2. We further evaluated the independent prognostic ability of NPC2 expression for overall survival (OS) and relapse-free survival (RFS) through the Kaplan–Meier curve and Cox analysis. The ability of NPC2 to predict OS and RFS was reflected by nomogram. Finally, the NPC2 expression was validated in the samples collected from patients and gastric cancer cell. The findings could provide new insights into the clinical significance of NPC2 expression in gastric cancer and its potential as a diagnostic and prognostic biomarker.

## Methods

### Data collection

Data was obtained from TCGA database (https://www.cancergenome.nih.gov). The raw data was preprocessed to remove outliers and normalize the expression levels of NPC2. A total of 380 patients with primary gastric cancer were involved (Supplementary Table 1). The clinicopathologic characteristics and expression levels of NPC2 in gastric cancer were analyzed by Fisher’s exact test and chi-square test, and non-parametric rank sum test, respectively^14^. The study was approved by the Ethics Committee of the First Hospital of Shanxi Medical University, and all methods were performed in accordance with the relevant guidelines and regulations. The 30 patient samples were obtained with appropriate informed consent and processed with approval.

### Diagnostic value analysis

The diagnostic value of NPC2 expression was analyzed by plotting the ROC curve and calculating the area under the curve (AUC). The AUC was used to determine the accuracy of NPC2 in diagnosing gastric cancer.

### Survival analysis

The Kaplan–Meier curve was used to evaluate the prognostic value of NPC2 expression for OS and RFS^15^. The log-rank test was used to compare the survival curves between different subgroups.

### Univariate and multivariate Cox analysis

Univariate Cox analysis was performed to assess the univariate association between NPC2 expression and survival. Multivariate Cox analysis was performed to evaluate the independent prognostic ability of NPC2 expression after adjusting for other potential confounding factors.

### Nomogram analysis

The nomogram was used to reflect the ability of NPC2 to predict OS and RFS. The concordance index (C-index) was calculated to assess the predictive accuracy of the nomogram^16^.

### GSEA analysis

The online GSEA analysis was performed after searching the TCGA for evaluation of NPC2 expression and enriched pathways^17^.

### Real-time quantitative PCR

The RT-qPCR was performed using the kit (Invitrogen, Thermo Fisher, USA) according to the instruction. 2^−ΔΔCt^ method was used for quantification. The NPC2 primers (forward primer: 5′-TGT GGC TCA GTG GCT TAG G-3′; reverse primer: 5′-CCA GGA AGG GAT TTC ACA CA-3′) and β-actin primers (forward primer: 5′-ACC CCA AAG CCA ACA GA-3′; reverse primer: 5′-CCA GAG TCC ATC ACA ATA CC-3′) were used.

### Immunohistochemistry

The immunohistochemistry (IHC) staining was performed as reported^18^. Anti-NPC2 primary antibody (MA5-29450, Thermo Fisher, USA) and corresponding rabbit secondary antibody were used.

### Cell culture and transfection

The cells were purchased from American Tissue Culture Collection and cultured in the necessary environment as reported^19^. There were four groups including si-NPC2 transfected group, NPC2 overexpressed group, si-NC transfected group as negative control, and un-transfected group as control. The validation of transfection efficiency was performed using qRT-PCR and western blot.

### Western blot

To confirm NPC2 knockdown, Western blot analysis was performed as previously reported^20^. Primary antibody NPC2 (sc-30346, Santa Cruz Biotechnology, USA) and goat secondary antibody (sc-2354, Santa Cruz Biotechnology, USA) were used.

### Cell proliferation assay

The cell proliferation assay was performed by CCK-8 in AGS and SGC-7901 cell as reported^20^. After plasmid transfection, the cells were cultured for 24 h. Then, the CCK-8 reagent (10 μL) was added and kept for 20 min. The absorbance at 490 nm was measured.

### Statistical analysis

All statistical analyses were performed using the R software (version 3.5.2). The Wilcoxon rank sum and the Kruskal–Wallis test was used for comparing two and multiple groups, respectively. A two-sided P value of < 0.05 was considered statistically significant.

## Results

### Characteristics of patients with gastric cancer

The characteristics of patients with gastric cancer were evaluated in both groups according to high and low NPC2 expression (Supplementary Table 2). The histologic grade (*P* = 0.048), T classification (*P* = 0.0081), and vital status (*P* = 0.018) were significantly different in two groups. At the same time, there were no statistical differences in age (*P* = 0.8347), gender (*P* = 0.4134), histological type (*P* = 0.0594), stage (*P* = 0.2087), N classification (*P* = 0.198), M classification (*P* = 0.7266), radiation therapy (*P* = 0.0581), and residual tumor (*P* = 0.62).

### NPC2 expression in gastric cancer

**Figure 1:**
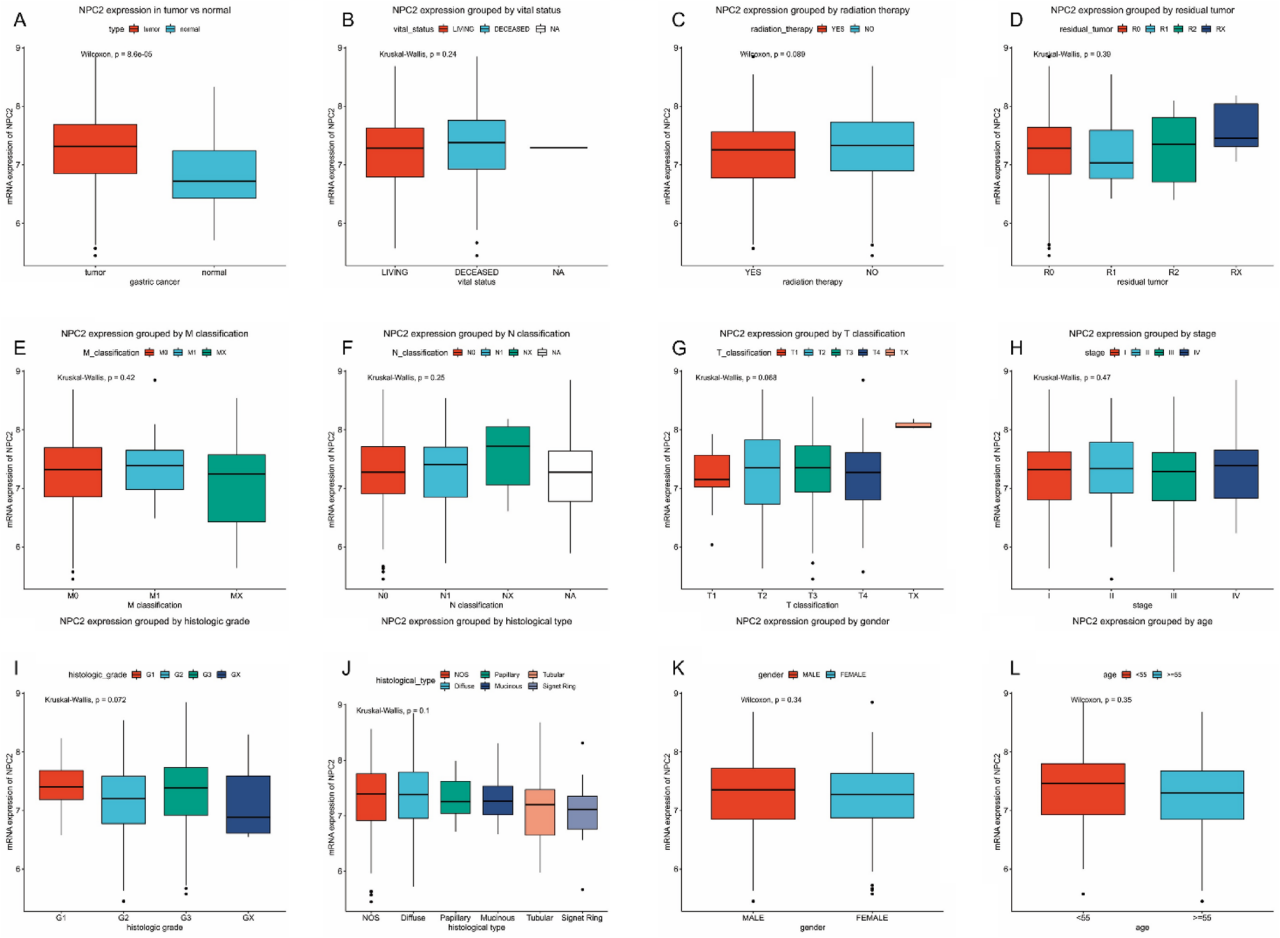
NPC2 expression in gastric cancer. (**A**) NPC2 expression in tumor versus paired normal tissue. NPC2 expression grouped by (**B**) vital status, **(C)** radiation therapy, (**D**) residual tumor, (**E**) M classification, (**F**) N classification, (**G**) T classification, (**H**) stage, (**I**) histologic grade, (**J**) histological type, (**K**) gender, and (**L**) age.

Compared with normal gastric tissue, the NPC2 expression was significantly higher (*P* < 0.001) in gastric cancer (Fig. 1A). Besides, the subgroup analysis was performed (Fig. 1B–L), including vital status, radiation therapy, residual tumor, TNM classification, stage, histological type, histologic grade, gender, and age. However, no statistical differences were found.

### Diagnostic value of NPC2 expression

The ROC curve analysis showed that NPC2 expression had a high diagnostic value for gastric cancer with AUC of 0.696 (Supplementary Figure 1A). Moreover, the AUC for stage I-IV was 0.660 (Supplementary Figure 1B), 0.721 (Supplementary Figure 1C), 0.678 (Supplementary Figure 1D), and 0.716 (Supplementary Figure 1E).

### High NPC2 expression is associated with poor OS and RFS

**Figure 2:**
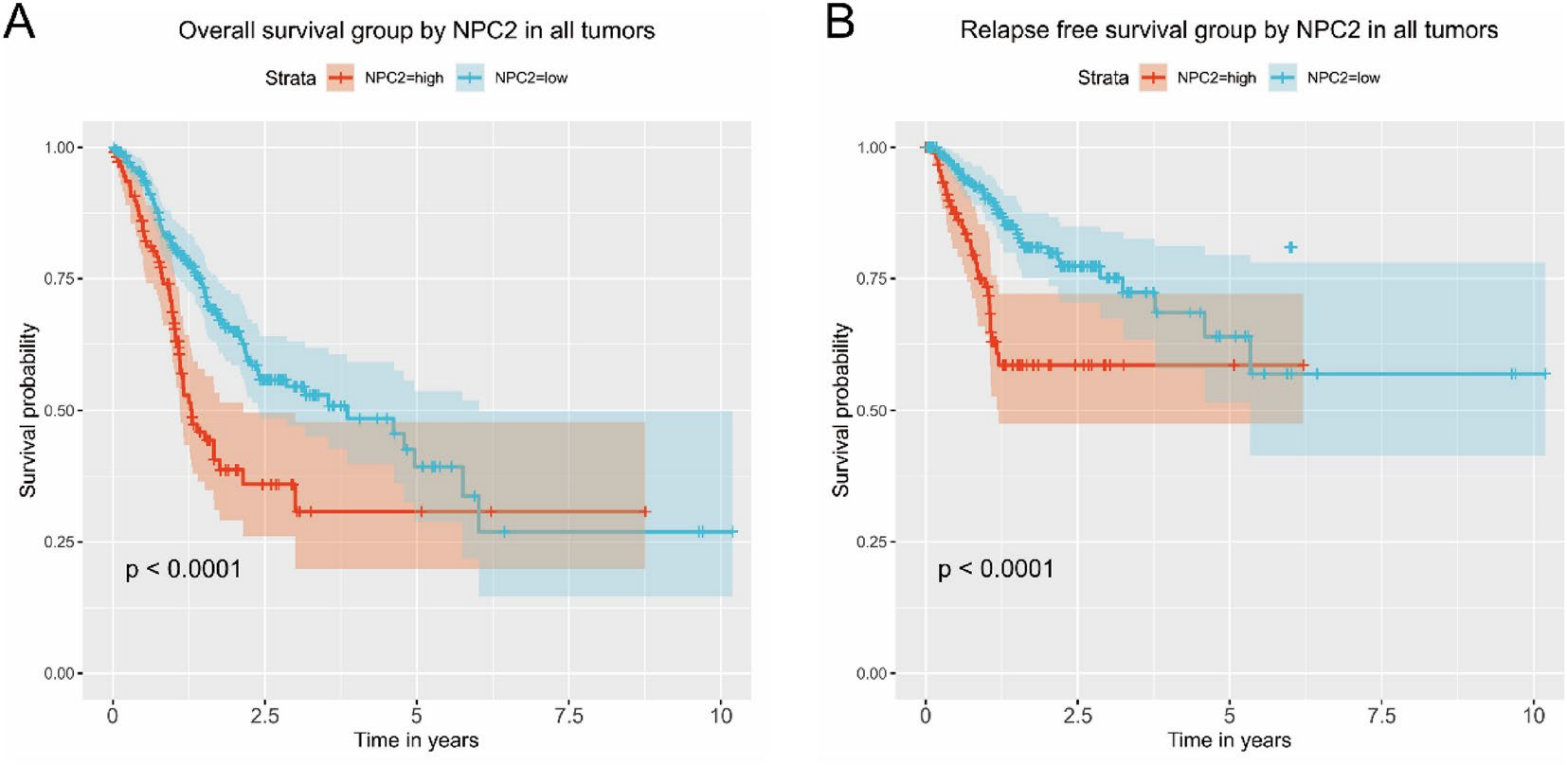
High NPC2 expression is associated with poor survival. (**A**) Overall survival. (**B**) Relapse free survival.

The Kaplan–Meier curves showed high NPC2 expression was significantly (*P* < 0.0001) associated with poor OS (Fig. 2A) and poor RFS (Fig. 2B).

### OS grouped NPC2 expression

**Figure 3:**
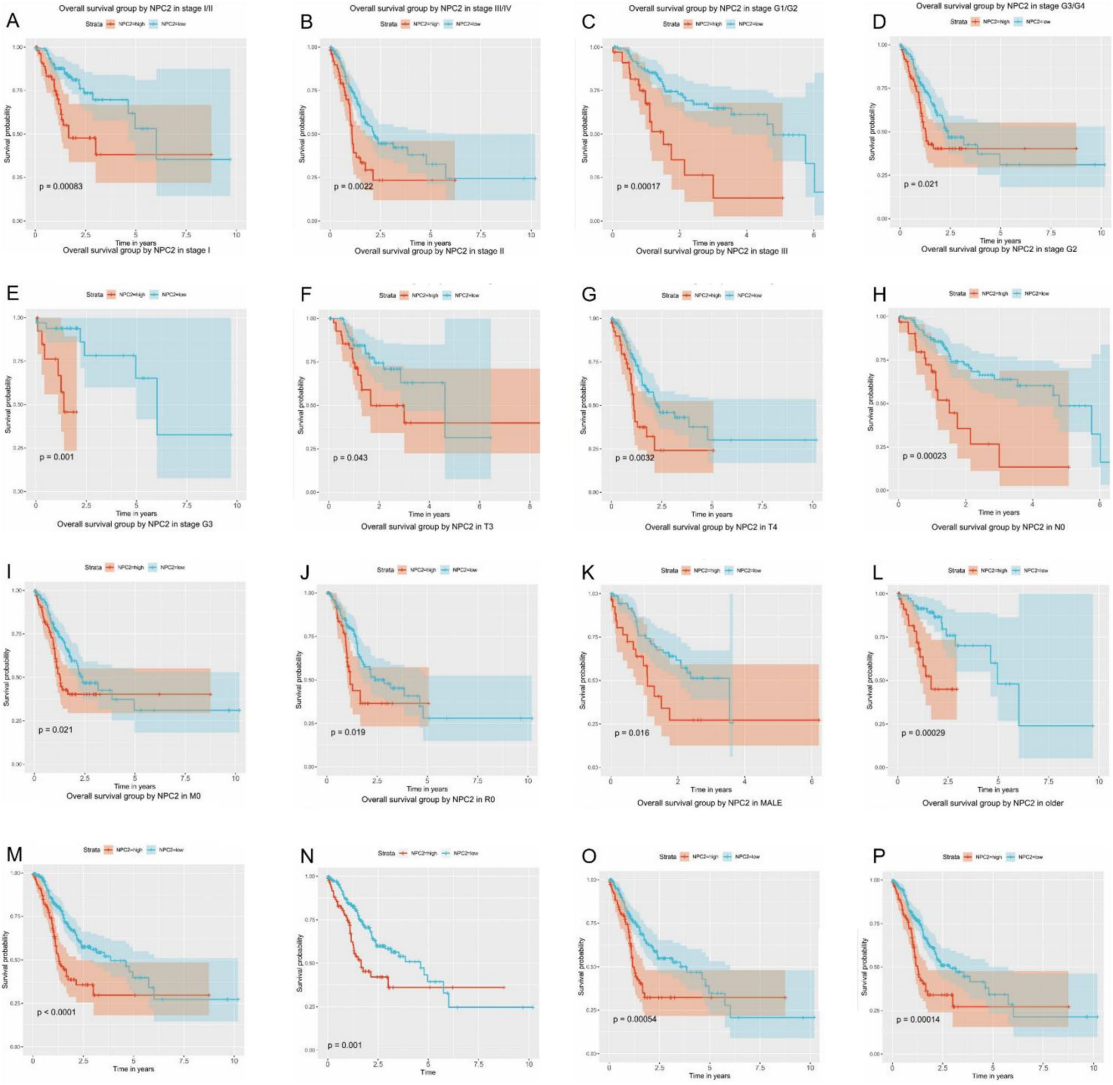
Overall survival grouped NPC2 expression. Overall survival group by NPC2 in (**A**) stage I/II, (**B**) stage III/IV, **(C)** G1/G2, (**D**) G3/G4, (**E**) stage I, (**F**) stage II, (**G**) stage III, (**H**) stage G2, (**I**) stage G3, (**J**) T3, (**K**) T4, (**L**) N0, (**M**) M0, (**N**) R0, (**O**) male, and (**P**) older.

The OS grouped NPC2 expression was significantly different in stage I/II, stage III/IV, stage G1/G2, stage G3/G4, stage I, stage II, stage III, stage G2, stage G3, T3, T4, N0, M0, R0, male, and older patients (Fig. 3A–P). Others were not significant (Supplementary Figure 2).

By univariate and multivariate analysis (Supplementary Figure 3A–B), the age [hazard ratio (HR): 1.825, 95% confidence interval (CI): 1.122–2.967, P = 0.015], stage (HR: 1.320, 95% CI: 1.049–1.661, *P* = 0.018), radiation therapy (HR: 0.471, 95% CI: 0.318–0.699, *P* < 0.001), residual tumor (HR: 1.695, 95% CI: 1.290–2.227, *P* < 0.001), and NPC2 expression (HR: 1.664, 95% CI: 1.190–2.327, *P* = 0.003) showed independent prognostic value for OS.

### RFS grouped NPC2 expression

**Figure 4:**
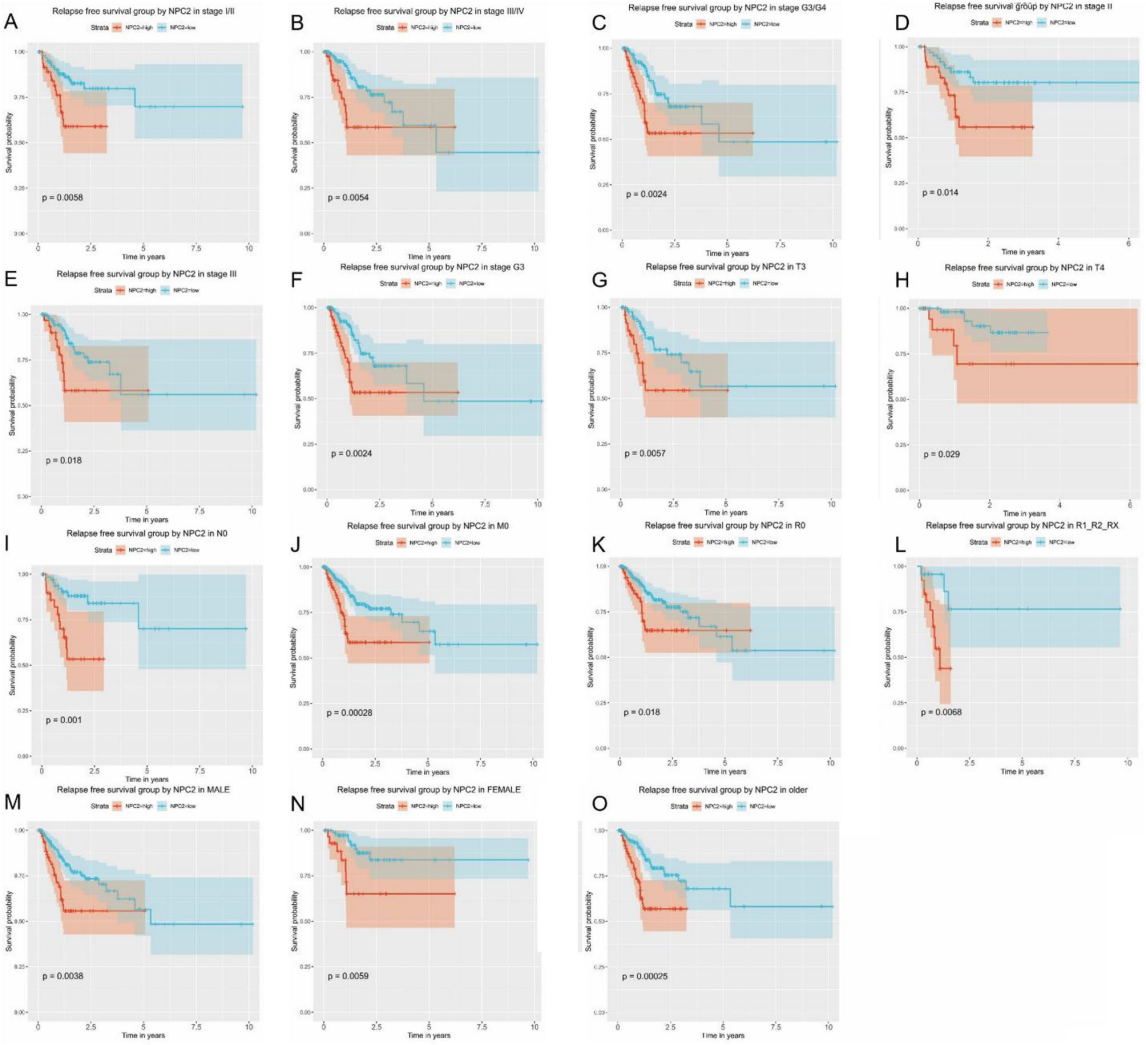
Relapse free survival grouped NPC2 expression. Relapse free survival group by NPC2 in (**A**) stage I/II, (**B**) stage III/IV, **(C)** G1/G2, (**D**) stage II, (**E**) stage III, (**F**) stage G3, (**G**) T3, (**H**) T4, (**I**) N0, (**J**) M0, (**K**) R0, (**L**) R1/R2/Rx, (**M**) male, (**N**) female, and (**O**) older.

The RFS grouped NPC2 expression was significantly different in stage I/II, stage III/IV, stage G3/G4, stage II, stage III, stage G3, T3, T4, N0, M0, R0, R1/R2, male, female, and older patients (Fig. 4A–O). Others were not significant (Supplementary Figure 4).

By univariate and multivariate analysis (Supplementary Figure 5A–B), the gender (HR: 1.960, 95% CI: 1.132–3.394, *P* = 0.016) and NPC2 expression (HR: 2.097, 95% CI: 1.289–3.412, *P* = 0.003) showed independent prognostic value for RFS.

### Predictive value of NPC2 expression in OS and RFS

**Figure 5:**
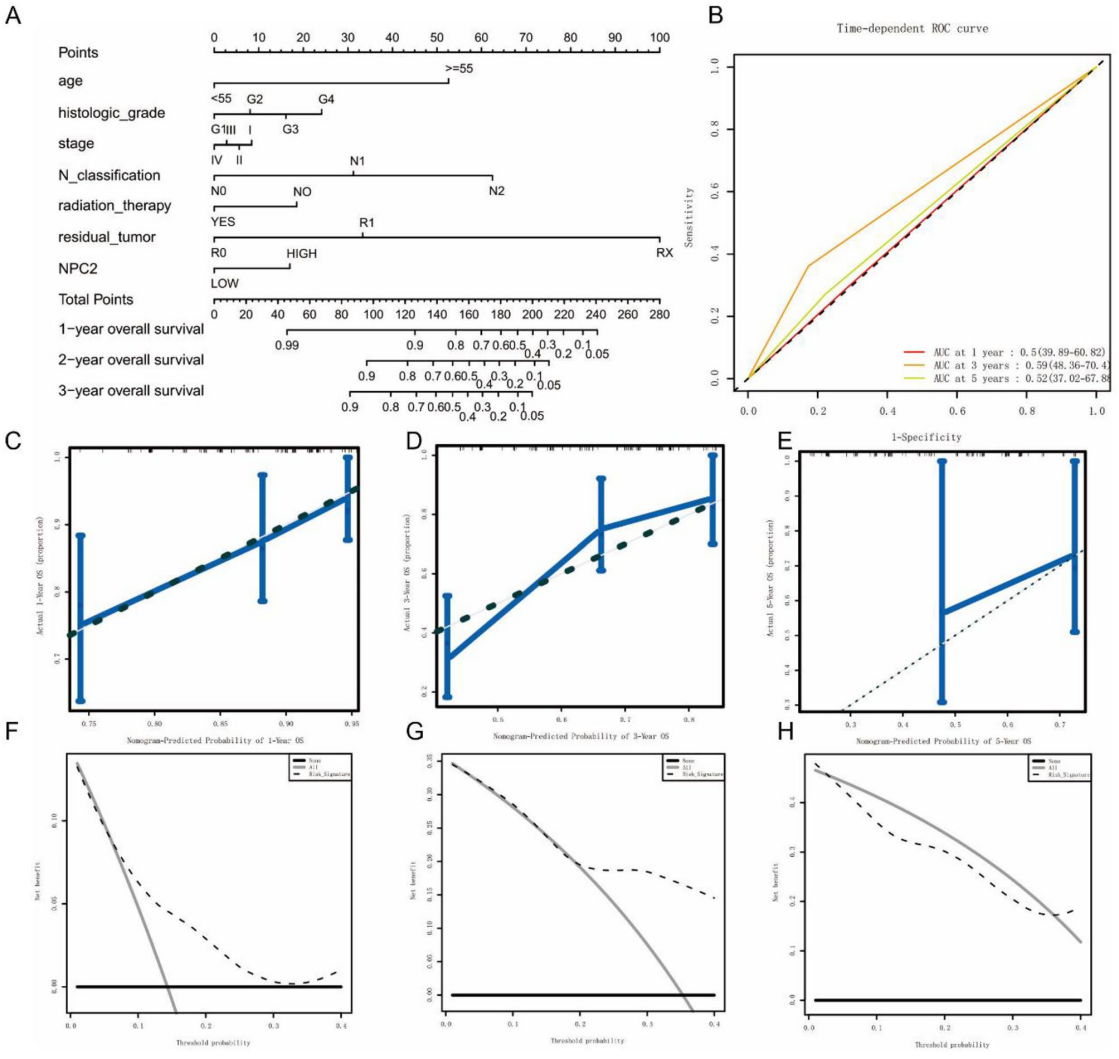
Predictive value of NPC2 expression in overall survival. (**A, B**) ROC curves evaluating the NPC2 expression for predicting overall survival. (**C–E**) Nomogram predicted 1-year, 3-year, and 5-year overall survival versus actual overall survival. (**F–H**) Decision curve analysis reflects the feasibility of NPC2 expression in predicting 1-year, 3-year, and 5-year overall survival.

**Figure 6:**
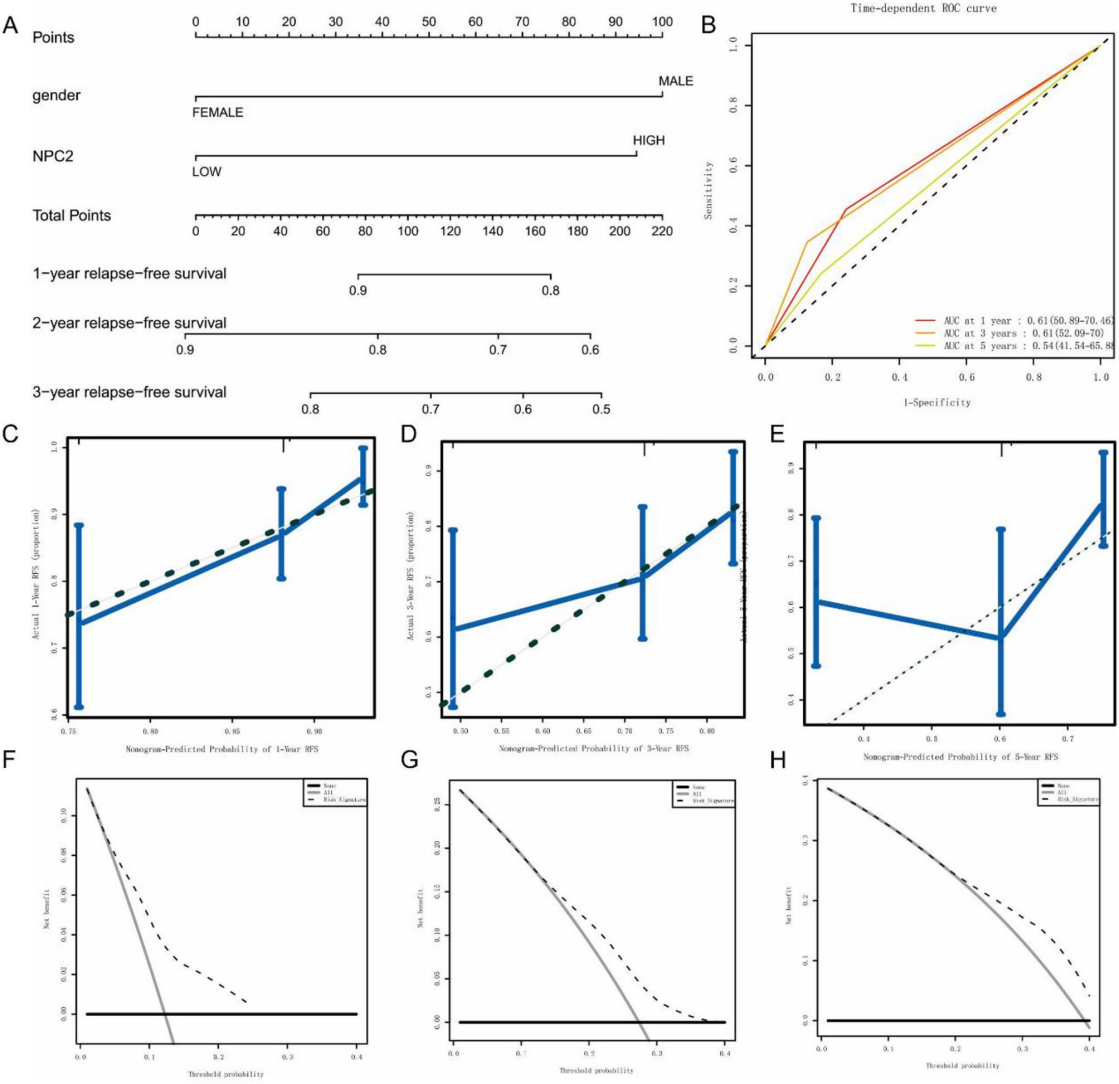
Predictive value of NPC2 expression in relapse free survival. (**A, B**) ROC curves evaluating the NPC2 expression for predicting relapse free survival. (**C–E**) Nomogram predicted 1-year, 3-year, and 5-year relapse free survival versus actual relapse free survival. (**F–H**) Decision curve analysis reflects the feasibility of NPC2 expression in predicting 1-year, 3-year, and 5-year relapse free survival.

The nomogram was used to further evaluate the ability of NPC2 to predict OS (Fig. 5A,B) and RFS (Fig. 6A,B). The results showed that NPC2 expression was a significant predictor of OS and RFS, with high predictive accuracy (Figs. 5C–H, 6C–H). Specifically, high NPC2 expression could predict shorter OS and RFS.

### High NPC2 expression-enriched pathways

**Figure 7:**
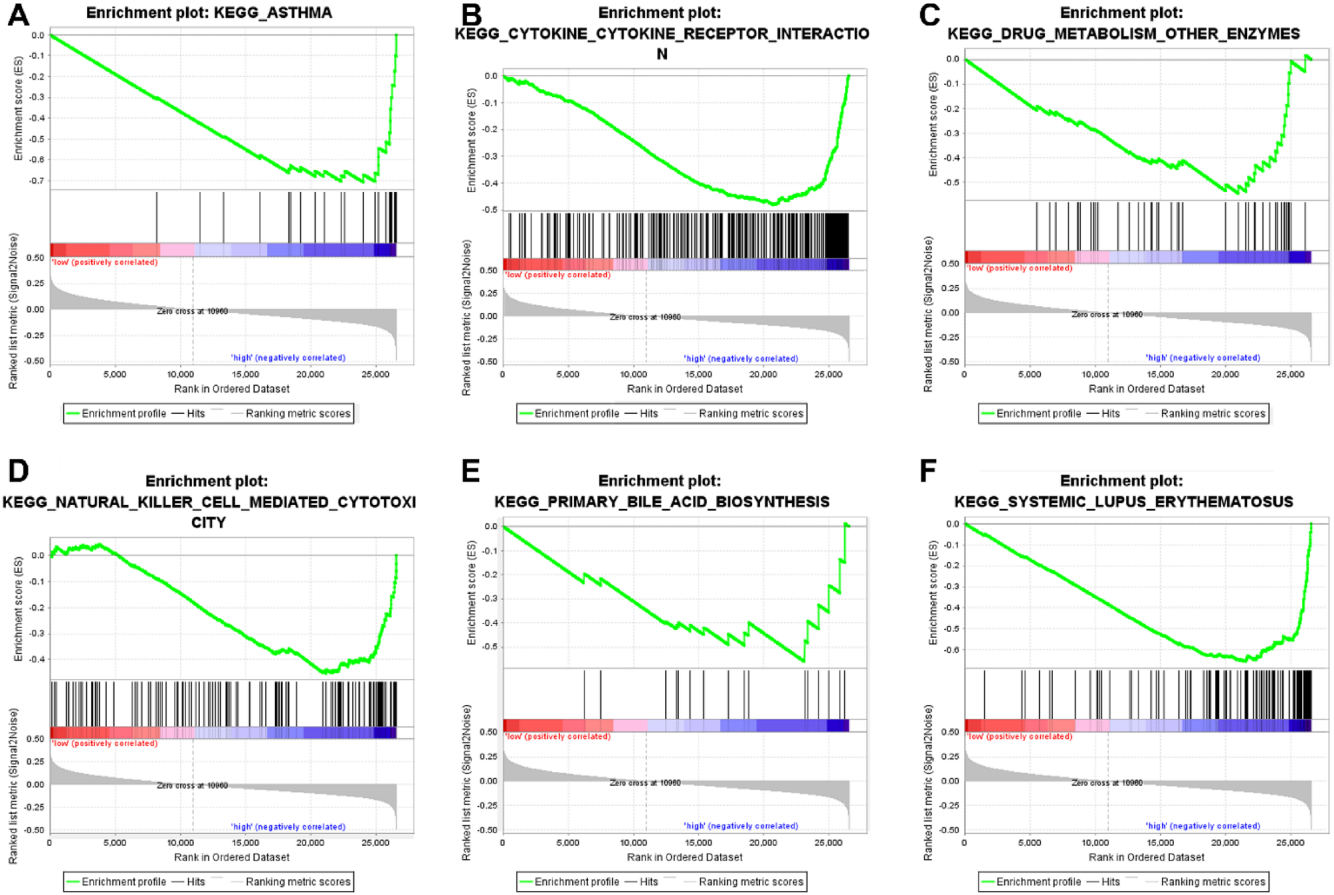
High NPC2 expression-enriched pathways. (**A**) Asthma. (**B**) Cytokine-cytokine receptor interaction. **(C)** Drug metabolism other enzymes. (**D**) Natural killer cell mediated cytotoxicity. (**E**) Primary bile acid biosynthesis. (**F**) Systemic lupus erythematosus.

The GSEA analysis (Supplementary Table 3) showed that high NPC2 expression was significant associated (*P* < 0.05) with asthma (Fig. 7A), cytokine-cytokine receptor interaction (Fig. 7B), drug metabolism other enzymes (Fig. 7C), natural killer cell mediated cytotoxicity (Fig. 7D), primary bile acid biosynthesis (Fig. 7E), and systemic lupus erythematosus (Fig. 7F).

### High NPC2 expression in tissue

**Figure 8:**
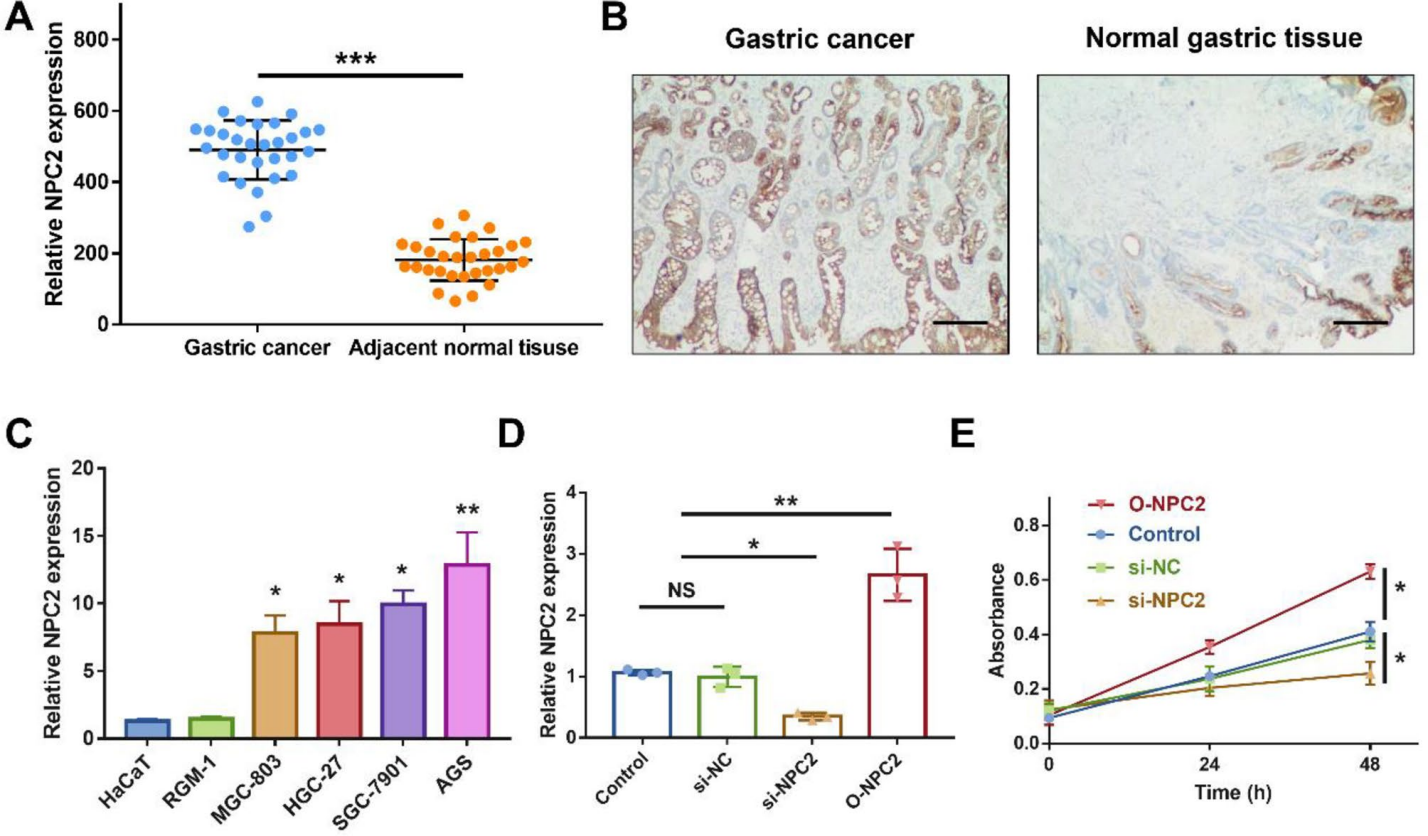
High NPC2 expression in tissue and cell. (**A**) NPC2 expression in 30 gastric cancer and adjacent normal tissues by qRT-PCR. (**B**) Representative IHC image of gastric cancer and normal gastric tissue. Scale bar = 100 μm. **(C)** Relative NPC2 expression in HaCaT, RGM-1, MGC-803, HGC-27, SGC-7901, and AGS by qRT-PCR. (**D**) Relative NPC2 expression in AGS cells transfected with control, si-NC, si-NPC2, and O-NPC2 by qRT-PCR. (**E**) Relative cell proliferation of AGS cell by CCK-8 assay. NS, no significance; *P < 0.05; **P < 0.01; ***P < 0.001.

The higher NPC2 expression was observed in gastric cancer, compared with adjacent normal tissue (*P* < 0.001, Fig. 8A). The IHC staining further confirmed the high NPC2 expression in gastric cancer (Fig. 8B). Moreover, the prognostic significance of NPC2 expression was validated by RT-PCR with another cohort consisting of 58 living patients and 32 deceased patients (Supplementary Figure 6). Higher NPC2 expression was associated with poor survival (*P* < 0.05).

### High NPC2 expression in cell

Besides, the NPC2 expression was evaluated in normal cells including HaCaT and RGM-1 (Human gastric epithelial cell), and gastric cancer cells including MGC-803 (Human low differentiated gastric cancer), HGC-27 (Human undifferentiated gastric cancer), SGC-7901 (Human gastric cancer with lymph node metastasis), AGS (Untreated human gastric adenocarcinoma cells). The NPC2 expression was higher in gastric cancer than normal cells (*P* < 0.05, Fig. 8C). Particularly, the NPC2 expression was highest in AGS, which was used for subsequent cell proliferation assay.

### NPC2 knockdown inhibits cell proliferation

First, the knockdown and overexpression efficiency were examined (Supplementary Figure 7 and Fig. 8D). The si-NPC2 treated AGS cell showed significant decreased NPC2 expression (*P* < 0.05), and O-NPC2 treated AGS cell showed significant increased NPC2 expression (*P* < 0.01). Then, the CCK-8 assay showed that NPC2 knockdown inhibits AGS cell proliferation while NPC2 overexpression promotes cell proliferation (*P* < 0.05, Fig. 8E). The involvements of NPC2 in proliferation was validated in SGC-7901 cell as well (Supplementary Figure 8).

## Discussion

Clinical stage at diagnosis directly determines the prognosis of patients with gastric cancer. Patients with localized, early-stage gastric cancer usually have a high 5-year overall survival rate (OS > 60%), whereas patients with local and distant metastases of gastric cancer have a significantly decreased 5-year OS of 30% and 5%, respectively^21^. Unfortunately, due to the occult nature and atypical nature of early clinical symptoms of gastric cancer, more than 60% of patients present with regional or distant metastasis at diagnosis^22^. Surgical resection is the best treatment option for patients with early-stage gastric cancer, and chemotherapy is the most important treatment for patients with unresectable or advanced metastasis. However, gastric cancer patients often respond poorly or even do not respond to chemotherapy due to their inherent or acquired resistance, becoming the most common cause of treatment failure. Therefore, the low rate of early diagnosis and chemoresistance are the main reasons for the poor prognosis of gastric cancer.

In the present study, we assessed the correlation between NPC2 expression and clinicopathologic characteristics in gastric cancer patients using data from The Cancer Genome Atlas (TCGA) database. Our findings indicated that NPC2 expression is significantly associated with certain clinicopathological features and can serve as a promising prognostic biomarker for gastric cancer patients.

Abnormal expression of NPC2 is closely associated with the progression of multiple malignant tumors. NPC2 expression is increased in patients with lung adenocarcinoma and their pleural effusions, but it is not clear whether NPC2 plays a role in lung adenocarcinoma^23^. The presence of NPC2 protein in pleural effusions of lung adenocarcinoma suggests that it may be used as a potential diagnostic marker for lung cancer. NPC2 overexpression correlates with the HER-2 subtype, suggesting that NPC2 upregulation may be a favorable prognostic predictor in breast cancer^24^. Notably, reduced expression of NPC2 was particularly observed in liver tumor tissues compared with normal counterpart tissues^25^. NPC2 is associated with lipid metabolism and the innate immune system, and it influences the formation and progression of various types of cancer cells, playing critical regulatory functions. Wei et al. found a relatively higher expression of NPC2 in glioblastoma and NPC2 overexpression inhibited cell proliferation^26^. Adachi et al. found silkworm NPC2 protein inhibits the growth of FM3A murine breast cancer cells^27^. Wang et al. reported that NPC2 overexpression decreased PDGF-BB-induced cell proliferation by inhibiting p38, JNK, and AKT phosphorylation^28^. Yet, the mechanism underlying the involvements of NPC2 in proliferation has reached no consensus.

The TCGA database involving huge data of gene related expression profile is keeping updated, and the number of patients may vary slightly at different time points. Further studies with larger sample sizes and longer follow-up periods are needed to validate our results and to determine the optimal cutoff value for NPC2 expression in gastric cancer patients. Additionally, it is essential to evaluate the feasibility and accuracy of NPC2 expression as a diagnostic and therapeutic target in gastric cancer.

## Conclusion

In conclusion, our findings provide evidence that NPC2 expression may serve as a promising prognostic biomarker for patients with gastric cancer. This information could have important implications for the early diagnosis, therapeutic strategies and personalized management of gastric cancer. The findings of this study can improve the therapy of GC patients by providing a novel therapeutic target and basis for further drug development.

### Supplementary Information

Supplementary Information.

## Data Availability

The data were obtained from public database. The experiment data are available at reasonable request.
