# Supplementary material for: Prognostic significance of high NPC2 expression in gastric cancer
Source: Sci Rep. 2023 Nov 24;13:20710. doi: 10.1038/s41598-023-47882-3 (PMC10673825; doi:10.1038/s41598-023-47882-3)
Supplement: Supplementary file 1 — Supplementary Information. [file 41598_2023_47882_MOESM1_ESM.pdf]

Supplementary information

Prognostic Significance of High NPC2 Expression in Gastric Cancer

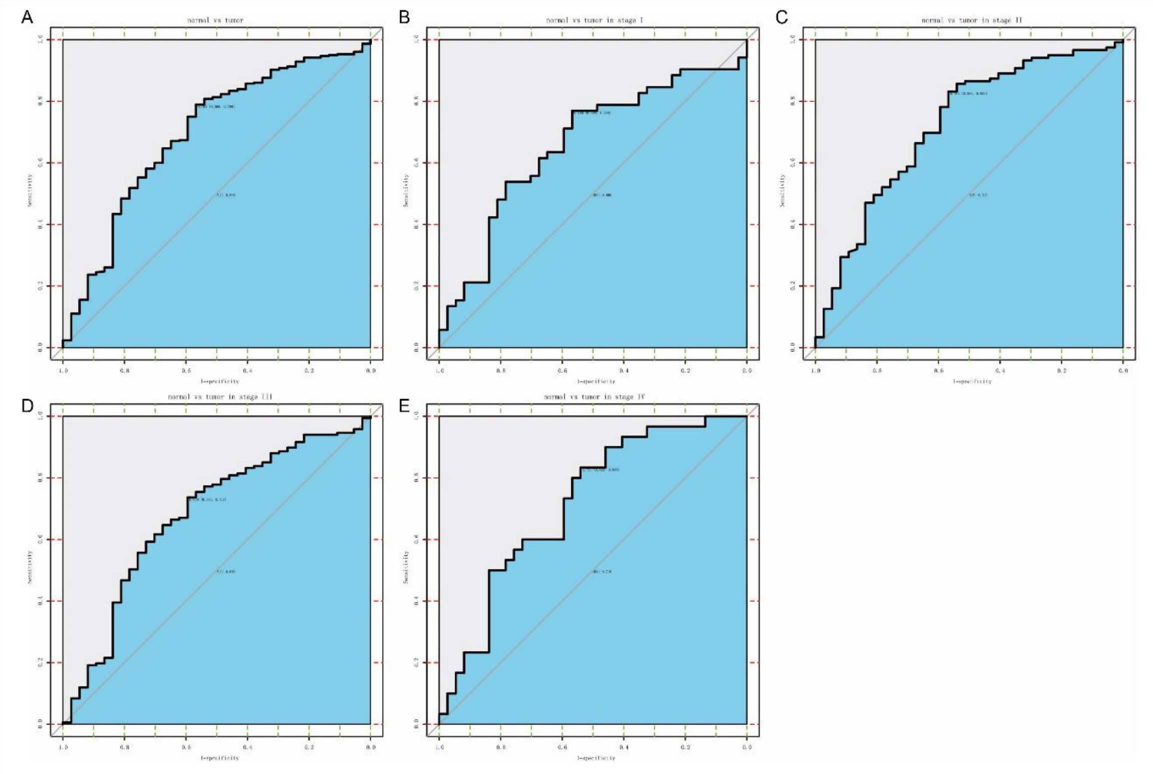

Figure 1 Diagnostic value of NPC2 expression. ROC curve in (A) normal vs. tumor, and (B) stage I, (C) stage II, (D) stage III, and (E) stage IV.

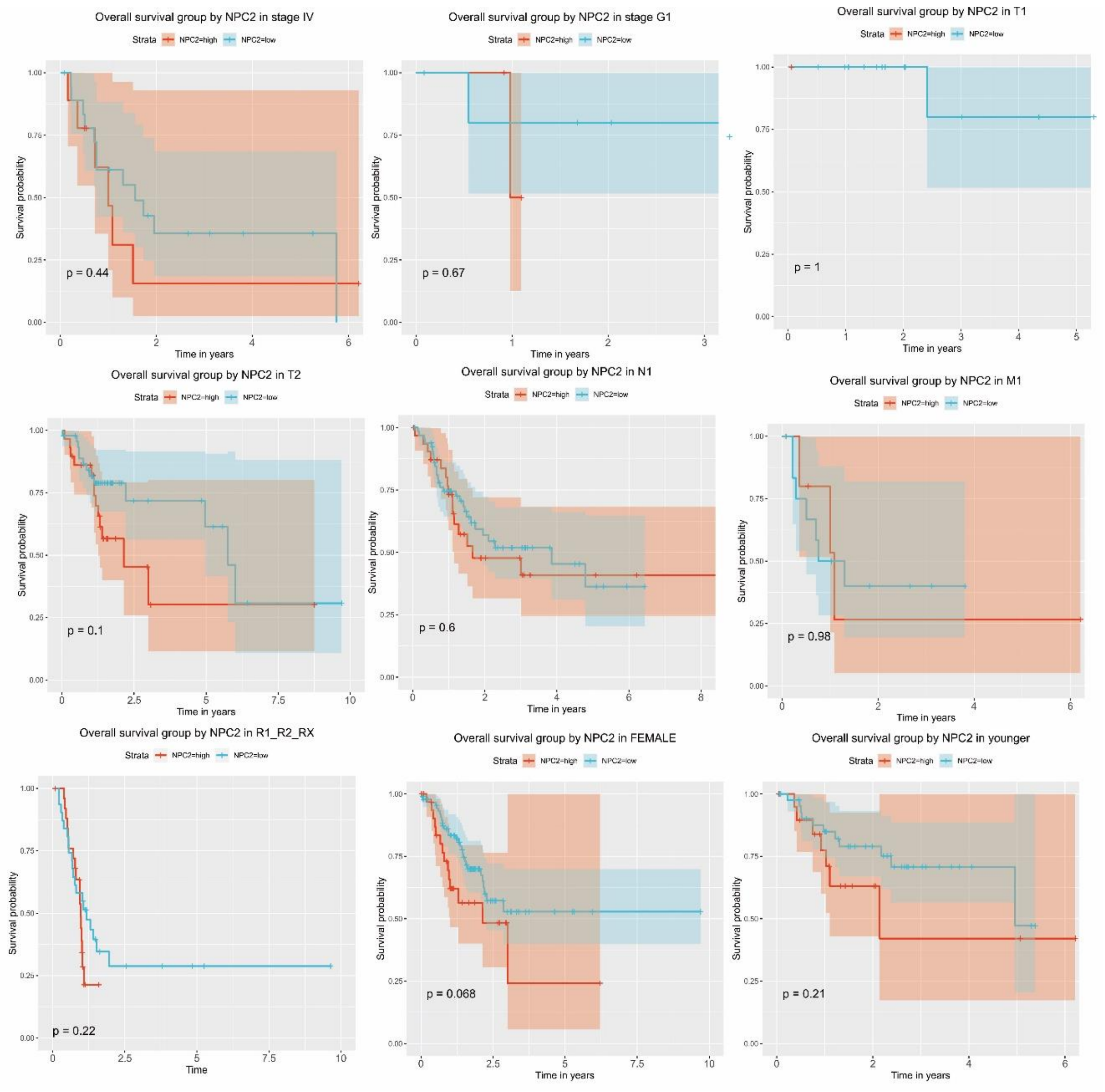

Figure 2 Overall survival grouped NPC2 expression.

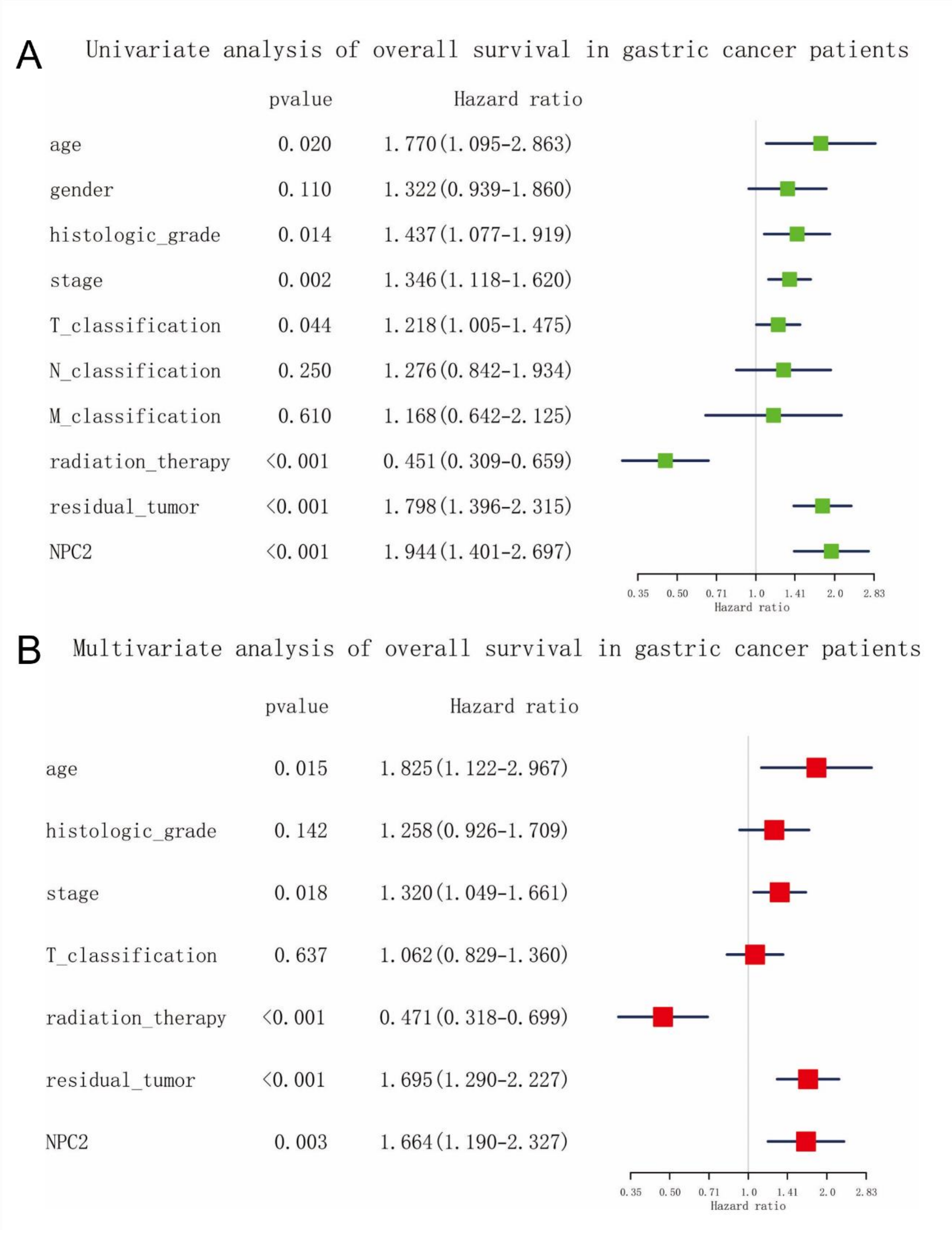

Figure 3 Cox analysis of overall survival. (A) Univariate analysis. (B) Multivariate analysis.

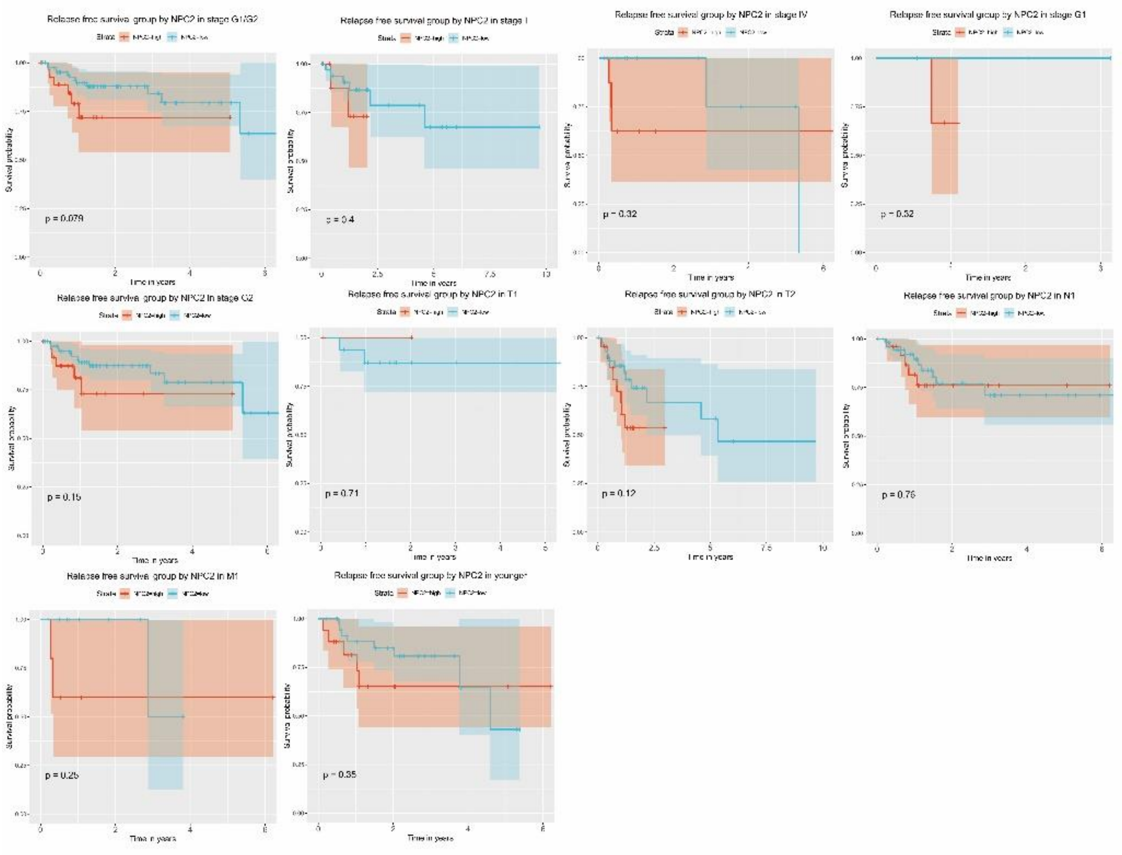

Figure 4 Relapse-free survival grouped NPC2 expression.

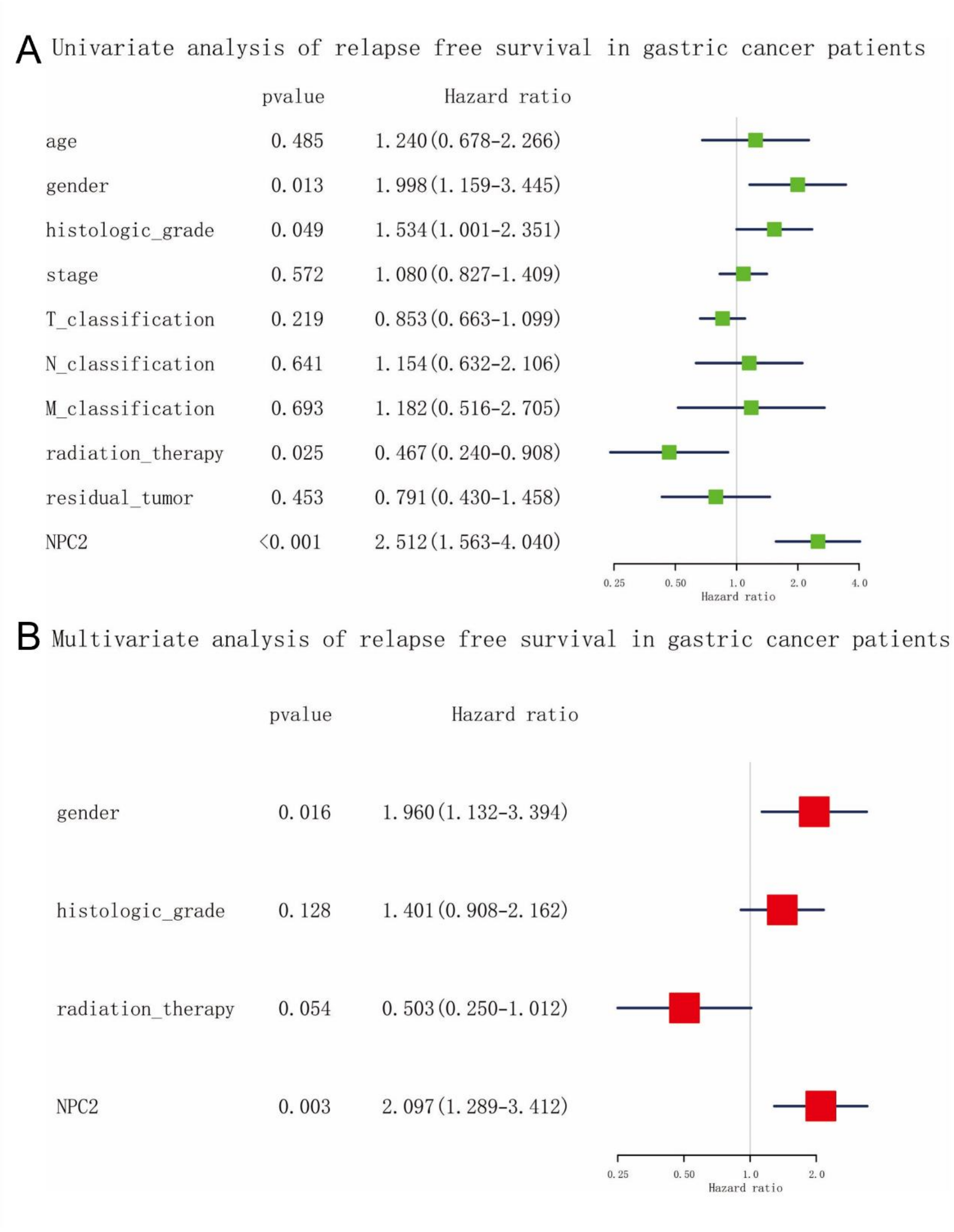

Figure 5 Cox analysis of relapse free survival. (A) Univariate analysis. (B) Multivariate analysis.

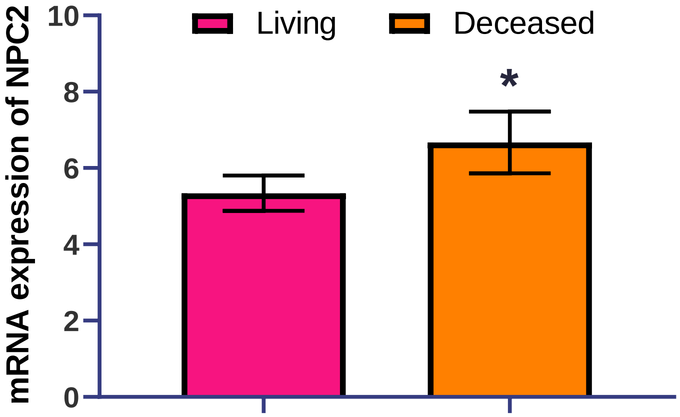

Figure 6 Prognostic significance of NPC2 expression validated by RT-PCR. Another cohort consisting of 58 living patients and 32 deceased patients (by retrospectively collecting the 5-year survival and examining mRNA expression of NPC2 in the reserved tumor) was analyzed. N = 90. *P < 0.05.

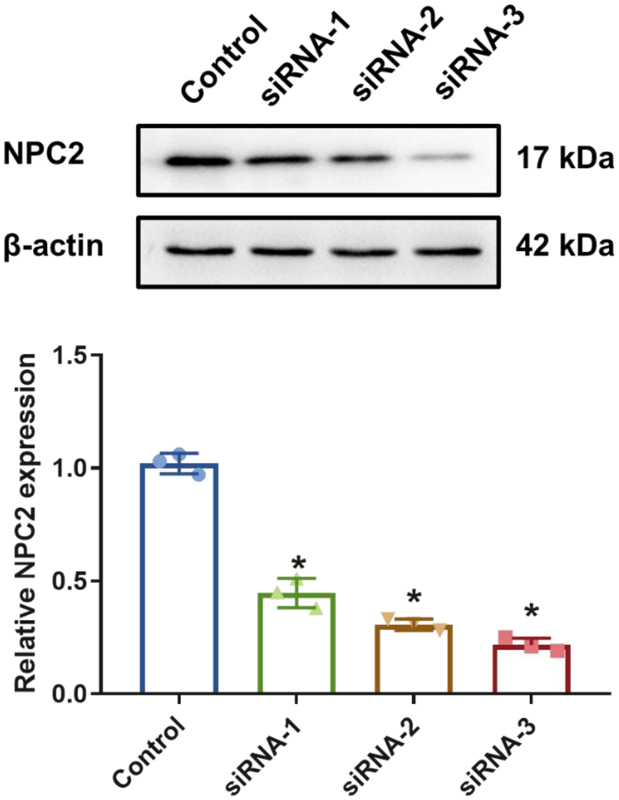

Figure 7 NPC2 knockdown by siRNA confirmed by RT-PCR (bottom panel) and western blot (top panel).

siRNA-3 (stated as si-NPC2) was used in all the subsequent experiments.

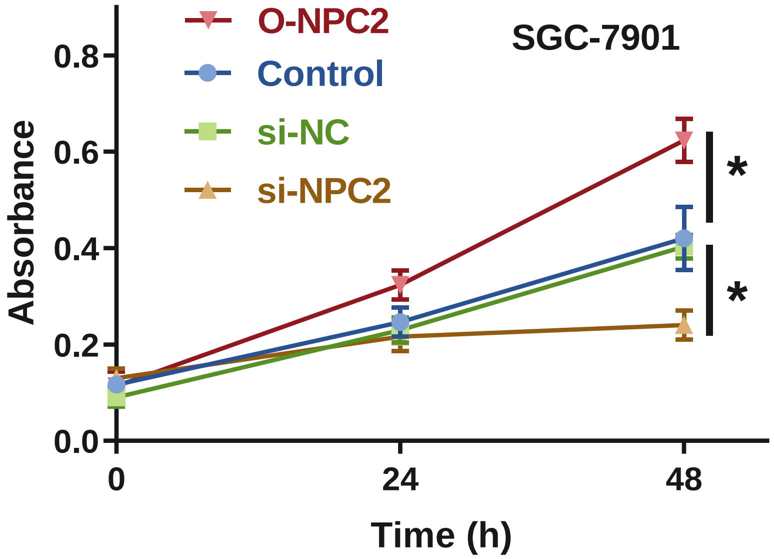

Figure 8 Relative cell proliferation of SGC-7901 cell by CCK-8 assay. *P < 0.05.

Table 1 General information of patients

Characteristics	Numbers of cases(%)
age	
<55	64(17.07)
>=55	311(82.93)
gender	
FEMALE	131(34.47)
MALE	249(65.53)
histological_type	
NA	1(0.26)
Diffuse	64(16.84)
Mucinous	19(5)
NOS	202(53.16)
Papillary	7(1.84)
Signet Ring	12(3.16)
Tubular	75(19.74)
histologic_grade	
G1	10(2.63)
G2	138(36.32)
G3	223(58.68)
GX	9(2.37)
stage	
NA	12(3.16)
I	52(13.68)
II	119(31.32)
III	167(43.95)
IV	30(7.89)
T_classification	
T1	21(5.53)
T2	77(20.26)
T3	173(45.53)
T4	106(27.89)
TX	3(0.79)
N_classification	
NA	1(0.26)
N0	117(30.79)
N1	101(26.58)
N2	75(19.74)
N3	77(20.26)

NX	9(2.37)
M_classification	
M0	341(89.74)
M1	20(5.26)
MX	19(5)
radiation_therapy	
NA	22(5.79)
NO	289(76.05)
YES	69(18.16)
residual_tumor	
NA	29(7.63)
R0	318(83.68)
R1	16(4.21)
R2	10(2.63)
RX	7(1.84)
vital_status	
NA	1(0.26)
DECEASED	152(40)
LIVING	227(59.74)
sample_type	
Primary Tumor	380(100)
NPC2	
high	116(30.53)
low	264(69.47)

Table 2 Characteristics of patients with gastric cancer

Parameter		N	High	(%)	Low	(%)	χ^2	P
Age	<55	64	21	(18.1)	43	(16.6)	0.0435	0.8347
	>=55	311	95	(81.9)	216	(83.4)		
Gender	FEMALE	131	36	(31.03)	95	(35.98)	0.6689	0.4134
	MALE	249	80	(68.97)	169	(64.02)		
Histological type	Diffuse	64	21	(18.1)	43	(16.35)	10.2467	0.0594
	Mucinous	19	3	(2.59)	16	(6.08)		
	NOS	202	73	(62.93)	129	(49.05)		
	Papillary	7	2	(1.72)	5	(1.9)		
	Signet Ring	12	3	(2.59)	9	(3.42)		
	Tubular	75	14	(12.07)	61	(23.19)		
Histologic grade	G1	10	3	(2.59)	7	(2.65)	7.5314	**0.048**
	G2	138	31	(26.72)	107	(40.53)		
	G3	223	80	(68.97)	143	(54.17)		
	GX	9	2	(1.72)	7	(2.65)		
Stage	I	52	14	(12.61)	38	(14.79)	4.5685	0.2087
	II	119	44	(39.64)	75	(29.18)		
	III	167	43	(38.74)	124	(48.25)		
	IV	30	10	(9.01)	20	(7.78)		
T classification	T1	21	2	(1.72)	19	(7.2)	13.9277	**0.0081**
	T2	77	29	(25)	48	(18.18)		
	T3	173	54	(46.55)	119	(45.08)		
	T4	106	28	(24.14)	78	(29.55)		
	TX	3	3	(2.59)	0	(0)		
N classification	N0	117	37	(32.17)	80	(30.3)	5.9283	0.198
	N1	101	32	(27.83)	69	(26.14)		

	N2	75	16	(13.91)	59	(22.35)		
	N3	77	25	(21.74)	52	(19.7)		
	NX	9	5	(4.35)	4	(1.52)		
M classification	M0	341	106	(91.38)	235	(89.02)	0.8569	0.7266
	M1	20	6	(5.17)	14	(5.3)		
	MX	19	4	(3.45)	15	(5.68)		
Radiation therapy	NO	289	95	(87.16)	194	(77.91)	3.5911	0.0581
	YES	69	14	(12.84)	55	(22.09)		
Residual tumor	R0	318	89	(89)	229	(91.24)	1.4946	0.62
	R1	16	4	(4)	12	(4.78)		
	R2	10	4	(4)	6	(2.39)		
	RX	7	3	(3)	4	(1.59)		
Vital status	DECEASED	152	57	(49.14)	95	(35.98)	6.1264	**0.0318**
	LIVING	227	59	(50.86)	168	(63.64)		

Note: X, unknown.

Table 3 High NPC2 expression-enriched pathways

NAME	SIZE	ES	NES	P value	Q value
Systemic lupus erythematosus	124	-0.65701	-1.99506	0.002092	0.17584
Drug metabolism other enzymes	42	-0.54511	-1.73932	0.011111	0.245262
Cytokine-cytokine receptor interaction	260	-0.48092	-1.63779	0.02686	0.245497
Asthma	25	-0.7078	-1.61531	0.043388	0.248443
Natural killer cell mediated cytotoxicity	126	-0.45246	-1.59506	0.047131	0.248043
Primary bile acid biosynthesis	16	-0.56215	-1.56087	0.043384	0.246022

The original, unprocessed version of gels and blots (Supplementary Figure 6) with multiple exposure images are shown as below:

Original-β-actin:

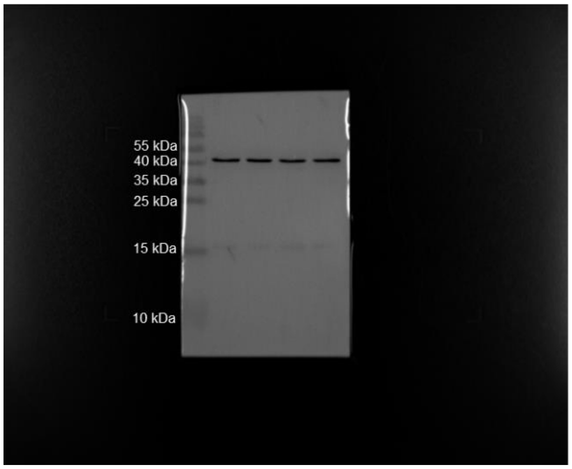

Original-NPC2:

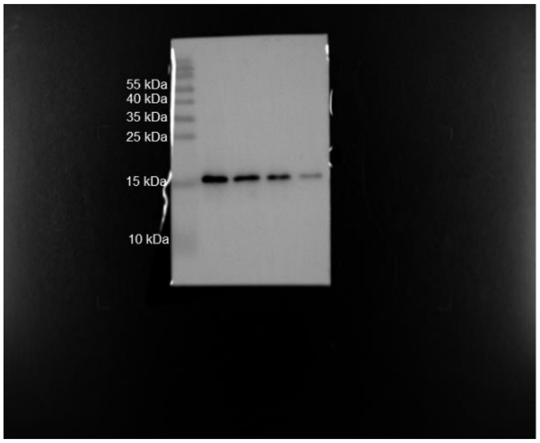

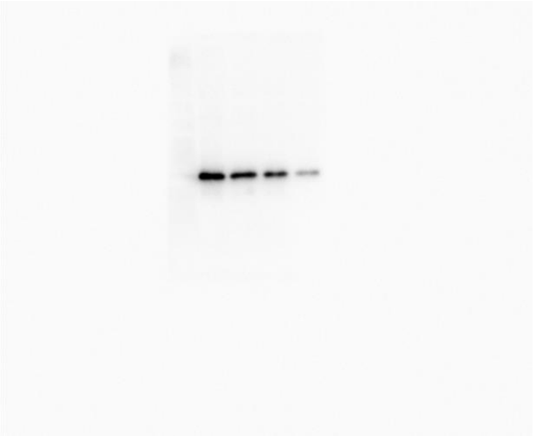

Note: As requested by the in-house editor, these are fuller-length, original, unprocessed blots performed with our samples for each antibody which confirms specific detection of the target antigen, with molecular size markings.
